# Endoscope-Assisted Evacuation of Acute-on-Chronic Subdural Hematomas: A Single-Center Series

**DOI:** 10.7759/cureus.27575

**Published:** 2022-08-01

**Authors:** Jorge F Urquiaga, Mayur S Patel, Najib El Tecle, Nabiha Quadri, Georgios Alexopoulos, Richard D Bucholz, Philippe J Mercier, Joanna M Kemp, Jeroen Coppens

**Affiliations:** 1 Neurological Surgery, Saint Louis University School of Medicine, St. Louis, USA; 2 Neurosurgery, Saint Louis University Hospital, St. Louis, USA; 3 Neurological Surgery, Saint Louis University, St. Louis, USA

**Keywords:** hematoma evacuation, trauma, hematoma, subdural, endoscopy

## Abstract

Purpose: Acute subdural hematomas are frequent, highly morbid, and affect all age groups. The most common mechanism of injury is a low-velocity fall, and the incidence of the disease is growing due to increasingly aggressive antithrombotic and anticoagulant therapies. In this study, we aimed to share our experience with the endoscopic-assisted evacuation of acute subdural hematoma, a less invasive procedure compared to standard craniotomy.

Methods: We retrospectively reviewed data of all consecutive patients aged 18 years and older who underwent endoscopic-assisted evacuation of acute-on-chronic subdural hematoma at our institution from 2015 to 2019. Preoperative, intraoperative, postoperative, and follow-up data were collected and reported. Statistical tests were done using Python statistical packages.

Results: Of the 35 patients that underwent this procedure, 32 were 18 years and older. The median age was 69.5 years and 37.5% were female. Twenty patients (62.5%) were on antiplatelet therapy, and six patients (18.75%) were on anticoagulants upon presentation. A fall was the most common cause of trauma (71.88%). The median operative time was 107 minutes. The median length of stay in days and Glasgow Coma Scale (GCS) at discharge were 8.5 and 15, respectively. There were no surgical site infections or in-hospital mortality in this series. At the latest follow-up, the median GCS and modified Rankin Scale were 15 and 1, respectively.

Conclusion: Evacuation of acute-on-chronic subdural hematomas can be performed safely and efficiently via a smaller craniotomy and with the assistance of an endoscope. This may represent a less invasive alternative than standard craniotomy/craniectomy in selected patients.

## Introduction

Acute subdural hematomas are a frequent and highly morbid condition affecting all age groups [1-4]. Mortality and morbidity of this condition increase with age; mortality has been reported to be as high as 90% in the elderly population [1-3,5-7]. A low-velocity fall at home is the most common mechanism of injury [5]. Such falls can result in a life-threatening hematoma, especially in patients on antithrombotic therapy or anticoagulants [8,9]. The incidence of subdural hematomas is increasing secondary to a larger portion of the population undergoing more aggressive antithrombotic and anticoagulant regimens [9,10]. This prompted us to look for less invasive alternatives in the management of this pathology. Typically, acute subdural hematomas that are considered amenable to surgery are evacuated using an open craniotomy [4,11,12]. More recently, endoscope-assisted craniotomies have been described as a less invasive treatment for chronic subdural hematoma [13-16]. The technical advantages of the endoscope enable the surgeon to reduce blood loss, reduce the operative time, minimize the size of the craniotomy, and potentially enhance postoperative recovery. Recent publications suggest that endoscopic-assisted evacuation of acute subdural hematomas through a small craniotomy could be safe and feasible in ideal candidates [17-24]. We retrospectively reviewed our experience in endoscope-assisted acute and acute-on-chronic subdural hematoma evacuation in what represents the largest series on this subject.

## Materials and methods

We retrospectively reviewed the data of all consecutive patients who underwent endoscope-assisted evacuation of acute and acute-on-chronic subdural hematoma at our institution between 2015 and 2019. Data collection was approved by the Institutional Review Board of our institution with a waiver of patient consent. Patients with acute subdural hematomas, defined as a hematoma developed within three days from a low-velocity blunt trauma to the head, and patients with acute aggravation of more chronic subdural hematomas that met the following criteria were offered endoscope-assisted subdural hematoma evacuation: (1) time since injury more than six hours, (2) stable repeat head computed tomography (CT) after six hours from presentation scan, (3) Glasgow Coma Scale (GCS) equal or above 8 upon presentation, and (4) no other major intracranial injury. Acute subdural hematomas could be de novo or superimposed to a more chronic subdural hematoma as long as the injury occurred by the aforementioned mechanism in the specified time frame.

Patients that were under antiplatelet or anticoagulant therapy upon presentation received platelet transfusions, fresh frozen plasma, vitamin K, or prothrombin complex concentrate prior to surgery as needed and based on surgeon’s preference.

Surgical technique

**Figure 1:**
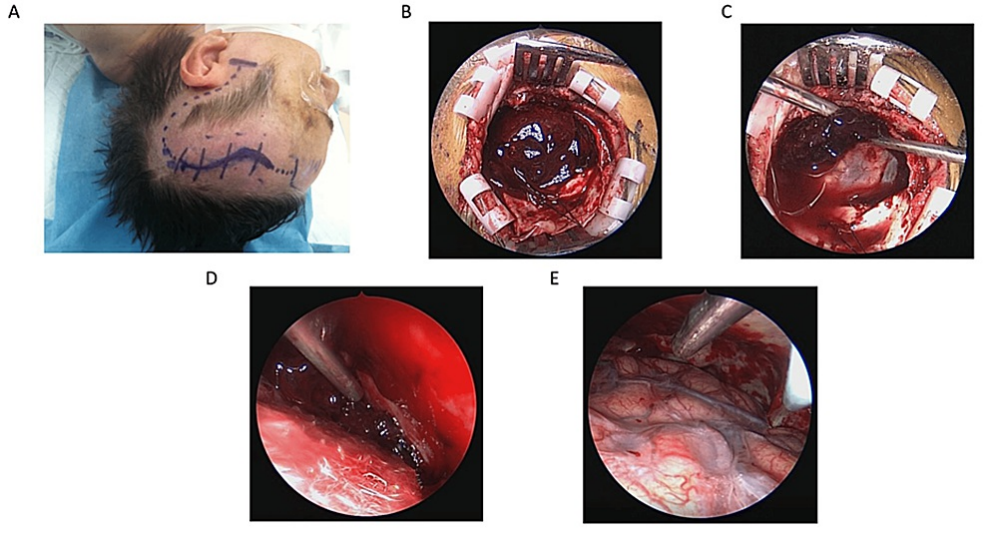
Endoscope-assisted subdural hematoma evacuation planning and intraoperative views (A) Incision planned along the superior temporal line. (B) Small ovoid craniotomy with opened dura and exposed acute subdural hematoma. (C) Evacuation of immediately visible hematoma. (D) In-depth evacuation of hematoma and membranes along the cerebral convexity toward the floor middle fossa. (E) Hematoma evacuation and hemostasis are achieved.

Patients were taken to the operating room and placed under general endotracheal anesthesia. They were positioned in the lateral decubitus position for unilateral procedures and in the supine position for bilateral procedures. The craniotomy was designed to allow optimal access to the entirety of the hematoma. The craniotomy was ovoid in all cases with 5 cm in the anteroposterior dimension and 3 cm in the cranial caudal dimension on average. It was typically centered along the superior temporal line and not the hematoma as long as the hematoma came to the superior temporal line. This allowed for maximal visualization with no brain retraction while using a 0° or 30° rigid endoscope (Karl Storz, Tuttlingen, Germany). The bone flap was elevated in standard fashion, and the dura was opened. Epidural tack-up sutures were placed on the dural edge to maximize visualization. The visible portion of the subdural hematoma was suctioned prior to introducing the endoscope. The remainder of the hematoma was then removed under direct endoscopic vision while working in a circumferential fashion. Membranes were peeled off when present, and good hemostasis was obtained. The cranioplasty and skin closure were completed in standard fashion. The decision of leaving a subdural drain was made on a case-to-case basis depending on the surgeon’s preference (see Figure 1 for details).

Recurrence was defined as a new re-accumulation of subdural fluid on the operated side causing increased midline shift as compared to the immediate postoperative scan, with or without new onset of symptoms attributed to the collection.

Statistical analysis

A descriptive analysis was chosen as the best method for reporting data as this was a case series and the assumptions of parametric tests were not fulfilled in this study. Categorical variables were reported as frequencies and percentages, and continuous variables were reported as the median and standard deviation. All analyses were performed using the Python statistical package.

## Results

A summary of the study population demographics is shown in Table 1. A total of 35 patients underwent surgery through this modality. Of these, 32 patients were older than 18 years and included in this analysis. The median age was 69.5 years old (95% CI, 67.42 to 71.58) and 37.5% were females. Of those included, 20 patients (63%) were on antiplatelet therapy, whereas six (19%) received anticoagulation therapy at the time of presentation. Five patients (15.63%) were on antiplatelet and anticoagulation therapies simultaneously upon presentation. A fall was the most common cause of trauma (72%); headache and focal motor deficit were the two most frequent presenting symptoms. The median baseline-modified Rankin Scale (mRS) and GCS were 0 and 15, respectively. Four patients (12%) had a GCS between 8 and 12 upon presentation. The median admission-to-surgery time was one day. The median preoperative midline shift was 7 mm. Seventy-eight percent of patients were found to have acute aggravation of a chronic subdural hematoma.

**Table 1:** Demographics and a summary of the patient population The mean age, sex, and the proportion of female patients are reported. Data regarding anticoagulation or antiplatelet therapy, the etiology of the hematoma, presenting symptoms, GCS at presentation to the emergency department, and the baseline-modified Rankin Scores are also reported. Distinctions of acute versus chronic hematoma and the number of days the patients were admitted preoperatively are stated. AMS: Altered mental status; mRS: Modified Rankin Score; GCS: Glasgow Coma Scale.

Characteristics	n (%)
Age (Median [CI 95%])	69.5 [67.42-71.58]
Sex (Female)	12 (37.5)
Antiplatelet/anticoagulation therapy	
Antiplatelet	20 (62.5)
Anticoagulation	6 (18.75)
Both	5 (15.63)
Cause	
Fall	22 (71.88)
Others	10 (28.12)
Presenting symptoms	
AMS	8 (25)
Aphasia	4 (12.5)
Focal motor deficit	10 (31.25)
Headache	10 (31.25)
Others	7 (21.88)
Baseline mRS (Median [CI 95%])	0 [0.00-0.76]
GCS at presentation	
GCS 13-15	28 (87.5)
GCS 8-12	4 (12.5)
Intubated at presentation	2 (6.25)
Age of hematoma	
Acute	7 (21.88)
Acute on chronic	25 (78.12)
Admission to surgery (days) (Median [CI 95%])	1 [0.77-1.23]
Preoperative midline shift (Median [CI 95%])	7 [6.34-7.66]

The surgical data is reflected in Table 2. The median operative time was 107 minutes (95% CI, 100.94 to 113.06). Right-sided craniotomies were performed in 62.5% of the cases; bilateral craniotomies were performed in two patients (6.25%). Active bleeding was only seen in two patients (6.25%), and the median estimated blood loss (EBL) was 100 cc. A subdural drain was left in 28 patients (87.5%). The surgical outcomes are described in Table 3. The postoperative midline shift median was 3.5 millimeters (95% CI, 2.99 to 4.01). The median length of stay (LOS) in days and GCS at discharge were 8.5 and 15, respectively. Seventeen patients (53.12%) were discharged to a rehabilitation facility, and seven (22%) were discharged to home. Only two patients (6.25%) were discharged to a long-term care facility (LTAC), and only one patient (3.13%) was discharged to hospice. In-hospital recurrence was seen in only one patient (3.13%) who was managed expectantly. The postoperative medical complications and seizure rates were 3% and 19%, respectively. There were no surgical site infections or in-hospital mortality in this series. The median latest postoperative outpatient follow-up was at 12 weeks after the procedure. At the latest postoperative follow-up visit, the median GCS and mRS were 15 and 1, respectively.

**Table 2:** Surgical demographic data The details of the surgery are reported, which include the mean operative time, site of the craniotomy, presence of active bleeding in patients, estimated blood loss, and the placement of a postoperative drain. NA: Not applicable.

Characteristics	Median (CI 95%)	n (%)
Operative time (min)	107 [100.94-113.06]	NA
Craniotomy side	NA	
Right		20 (62.50)
Left		10 (31.25)
Bilateral		2 (6.25)
Active bleeding	NA	2 (6.25)
Estimated blood loss (ml)	100 [85.46-114.54]	NA
Presence of postoperative drain	NA	28 (87.50)

**Table 3:** Postoperative outcomes and characteristics after the operation and during discharge Postoperative outcomes analyzed are the midline shift of imaging, the median number of days of having a drain placed, length of total hospital stay, and GCS at discharge. Additionally, the location of discharge and postoperative complications (including infection, medical complications, and seizures) and in-hospital mortality are noted. The GCS and modified Rankin Score at the first follow-up visit are also reported. GCS: Glasgow Coma Scale; SNF: Skilled-nurse facility; LTAC: Long-term acute care; mRS: Modified Rankin Score; NA: Not applicable.

Characteristics	Median (CI 95%)	n (%)
Postoperative midline shift (mm)	3.5 [2.99-4.01]	NA
Days with drain	2 [1.83-2.17]	
Length of stay (days)	8.5 [7.06-9.94]	
GCS at discharge	15	
Disposition	NA	NA
Home		7 (21.87)
Rehab		17 (53.12)
SNF		5 (15.63)
LTAC		2 (6.25)
Hospice		1 (3.13)
In-hospital recurrence	NA	1 (3.13)
Postoperative complications	NA	NA
Surgical site infection		0 (0)
Medical complications		1 (3.13)
Postoperative seizure		6 (18.75)
In-hospital mortality	NA	0 (0)
Outpatient follow-up (weeks)	12 [9.92-14.08]	NA
Follow-up GCS	15	NA
Follow-up mRS	1 [0.71-1.29]	NA

## Discussion

The results observed in our experience with endoscopic-assisted evacuation of acute and acute-on-chronic subdural hematoma suggest it is a safe and effective procedure for select patients. The use of the endoscope through a small craniotomy at the superior temporal line offers a unique opportunity to easily remove the hematoma and membranes. It is important to emphasize the inclusion criteria used in this study; the overall clinical status of the patients was noncritical, there was radiological evidence of clot stability, and no additional intracranial injuries were present. In addition to these, in our series, the mechanism of injury was mainly a low-velocity mechanical fall resulting in a head trauma, which could have played a role in the presence of an isolated acute subdural hematoma with a relatively reassuring neurological exam. Considering that the majority of the patients were older than 65 years old, advanced age could have facilitated the use of this technique due to age-related brain atrophy allowing more space to work with the endoscope and surgical instruments within the narrow operating field. All these factors potentially made the likelihood of facing postoperative brain edema lower and allowed for the planning of this less invasive procedure.

The proposed surgical technique was performed safely despite 75% of the patients being on antithrombotic therapy or anticoagulation at the time of presentation. Both were reversed prior to the surgical intervention; the reversal method was based on the surgeon’s preference and blood bank recommendations. The descriptive nature of this study does not allow for recommendations on a standardized antithrombotic or anticoagulation reversal protocol.

The median operative time in our study of 107 minutes was similar to that reported in the previous series of endoscopic-assisted subdural hematoma evacuations found in the literature [19-21,23,24]. There will definitely be a learning curve for neurosurgeons unfamiliar with the use of the endoscope in their daily practice that will impact the operative time at first, but as with any other newly learned technique, continuous use will likely improve surgical procedure duration. The operative time for standard craniotomy/craniectomy for acute subdural hematoma evacuation reported in the literature has a wide range of reported times as short as 49 minutes to longer than two hours [21,25-27].

**Figure 2:**
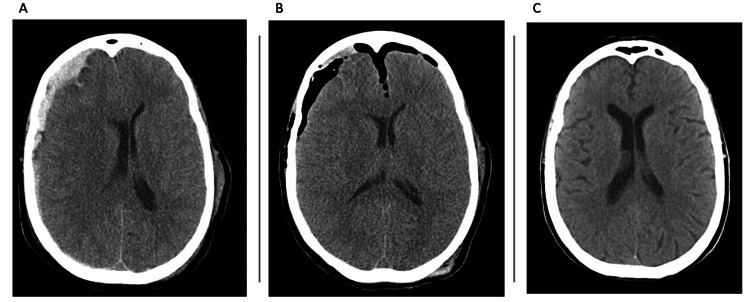
Preoperative and postoperative computed tomography scans of the same patient with subdural hematoma (A) Axial view of a computed tomography (CT) scan depicting an acute subdural hematoma of a patient from our series. (B) Axial view of a CT scan of the same patient in the immediate postoperative phase. (C) Axial view of a CT scan of the same patient during a follow-up visit four weeks after surgery.

The median midline shift in our series decreased to 3.5 mm as compared to a preoperative median of 7 mm (see Figure 2). Our median LOS was 8.5 days, which was better compared to the only other series utilizing endoscopic-assisted evacuation with reported data on LOS (mean 23.4 days); however, the population in that study had a higher mean age and baseline mRS, and the GCS at presentation was lower [22]. The LOS in our series was also shorter than that in reported studies on the standard evacuation of acute subdural hematomas; nevertheless, a significant portion of the reported data on standard evacuation proceeds from severely ill patients in contrast to the selected patients in our study. In a large retrospective study published in 2012, Ryan et al. reported their findings on mortality and functional outcomes in adults presenting with acute traumatic subdural hematoma [28]. Within the group that underwent standard surgical evacuation, 49% of their population had a GCS of 13-15 and a mean LOS of 15.1 days. The average LOS reported from national databases of patients that underwent a standard craniotomy or craniectomy for evacuation of subdural hematomas is 10-13 days [7,29]. It is evident that patient selection is crucial for the success of this still novel technique.

Similar to other series [22-24], a subdural drain was left in the subdural space to allow for immediate postsurgical fluid re-accumulation in almost all cases. Only one patient in this series presented a recurrence. This patient had an acute subdural hematoma as a consequence of a mechanical fall, and his past medical history included end-stage renal disease on hemodialysis and alcoholic liver disease with baseline thrombocytopenia. This patient received platelet transfusion prior to surgery and did not have a subdural drain placed during surgery. Recurrence was found on a scan obtained on postoperative day 1 prior to extubating. The recurrence was managed expectantly. The patient deteriorated over the next several days after extubating due to pulmonary congestion and progressive neurological decline that led to a tracheostomy and percutaneous endoscopic gastrostomy. The patient was discharged to LTAC after a total LOS of 14 days.

The overall postoperative complication rate found in our series was low. Six patients (19%) had postoperative seizures; those same six patients had a GCS of 15 at their latest follow-up visit. Other studies have reported seizure rates between 15% and 25% in adults [3,30] and 40% in elderly patients during the acute postoperative phase after a standard craniotomy for acute subdural hematoma evacuation. Zero patients had a surgical site infection. Only one patient (3.13%) had a postoperative medical complication, which was a catheter-related urinary infection. These results are better than the reported data on standard craniotomy/craniectomy that go as high as 19% infection rate including urinary tract infections, ventilator-associated pneumonia, cellulitis, etc. [3,7,28].

Most of our patients (53%) were discharged to a rehabilitation facility. This is not unexpected since the median age was 69.5 years and at least 40% of the patients presented with a focal motor deficit and/or aphasia requiring further therapy. Nevertheless, 22% of the patients were discharged home. The median latest postoperative outpatient follow-up was 12 weeks. At follow-up, the median mRS was 1, reflecting that patients were able to continue to live independently.

Patients with acute-on-chronic subdural hematoma can be effectively treated with burr holes. However, oftentimes, the acute component of the collection can represent a challenge for successful evacuation via this method, given its consistency. Conversion of burr holes to a larger craniotomy due to this particular difficulty is not uncommon. In anticipation of this obstacle, the endoscope-assisted technique through a small craniotomy was widely preferred by the surgeons in our institution given its ample visibility and maneuverability, which facilitated access to distant clot remnants and provided direct verification of optimal hematoma evacuation. The craniotomy location at the superior temporal was a key to accessing the aforementioned distant clot remnants utilizing a rigid endoscope. We believe that proficient use of a rigid endoscope requires a shorter learning curve than the flexible one, and it can achieve a comparable evacuation if the craniotomy location is carefully planned. All the patients with acute-on-chronic subdural hematoma in this series had a clear history of head trauma within three days from the presentation, which supported our decision to consider them as part of this study. Direct comparisons between the endoscope-assisted and the burr holes techniques as well as rigid versus flexible endoscope techniques are beyond the scope of this study.

The endoscope-assisted technique has proven to be an asset for the evacuation of chronic subdural hematomas. The advantages of this technique described in numerous publications can certainly be applied for the evacuation of acute subdural hematomas in appropriately and highly selected patients that present after a low-velocity head trauma (e.g., ground-level fall) with a high GCS. In this study, we present our initial experience utilizing the endoscope-assisted technique in this patient population. It is of extreme importance to emphasize that the endoscope-assisted technique should not be the method of choice for the treatment of an acute subdural hematoma secondary to a high-velocity trauma in a patient presenting with a low GCS. The decompressive craniectomy and subdural hematoma evacuation shall remain the standard procedure for such patients.

Our study has several limitations. The retrospective design of this study does not allow for presurgical management control as well as standardized follow-up. Even though this is the largest sample on this specific technique for acute-on-chronic subdural hematoma evacuation in the literature, a larger, prospective, and controlled trial should take place in order to have recommendations with a high level of evidence as well as solid outcome data to permit proper comparison against well-established techniques.

## Conclusions

The evacuation of acute and acute-on-chronic subdural hematomas via a small craniotomy and with the assistance of an endoscope might represent a safe and efficient alternative in patients that meet the specific criteria that allow this less invasive procedure to be planned. Further studies in a more controlled setting and with a larger population are needed to compare its efficacy against the standard craniotomy as well as to possibly expand its indications.
